# Mycobacterium nonchromogenicum bacteremia in an AIDS patient.

**DOI:** 10.3201/eid0401.980119

**Published:** 1998

**Authors:** J Mayo, J Collazos, E Martínez


8. Children's mental health research severely neglected, says National Institute of Mental Health Director. Washington Fax [serial online] 1997 Apr 8 [cited 1998 Jan 5]. Available from URL: http://www.washington-fax.com

### *Mycobacterium nonchromogenicum* Bacteremia in an AIDS Patient

**To the Editor:** *Mycobacterium avium* complex is the most common nontuberculous mycobacterium that causes disseminated infection in HIV-positive patients (1). Other less common nontuberculous mycobacteria responsible for disseminated disease in these patients are *M. fortuitum* (2), *M. genavense* (3), *M. gordonae* (4), *M. haemophilum* (5), *M. kansasii* (6), *M. malmoense* (7), *M. marinum* (8), *M. scrofulaceum* (9), *M. simiae* (10), *M. szulgai* (11), *M. terrae* (9), and *M. xenopi* (2,9). Although only *M. genavense*, *M. kansasii*, and *M. xenopi* are significantly more frequent in these patients (2,3,9), HIV infection is likely a predisposing condition for all nontuberculous mycobacterial infections. We report the first case of disseminated infection caused by *M. nonchromogenicum* in an HIV-infected patient.

A 28-year-old man with HIV infection acquired by sharing injection tools was seen in our outpatient clinic because of intermittent fever, drenching nocturnal sweats, and cough with purulent sputum of 4 months' duration. He also reported a weight loss of 10 kg in the previous 2 months. He had been diagnosed with bronchial infection in another hospital and had been treated with an unknown antibiotic. After this treatment, respiratory symptoms had improved somewhat, but fever and constitutional symptoms continued. His only previous opportunistic infection had been recurrent oral and esophageal candidiasis. The last CD4-cell count had been 16/µL 1 year earlier, and he was receiving didanosine and prophylactic therapy with cotrimoxazole and fluconazole. On physical examination the patient appeared ill; he was febrile, cachectic, and had thrush and oral hairy leukoplakia. Neither lymphadenopathy nor abnormal cardiopulmonary symptoms were found. The liver, which had enlarged since the last examination, was palpated 6 cm below the right costal margin. Abnormal laboratory values included aspartate aminotransferase 61 U/L, gamma-glutamyl transferase 209 U/L, lactate dehydrogenase 516 U/L, hemoglobin 12.3 g/dL, leukocyte count 4,300/µL (66% neutrophils, 19% band forms, 1% metamyelocytes, 3% lymphocytes, 11% monocytes), platelet count 130,000/µL, and erythrocyte sedimentation rate 72 mm/h. Chest X-rays were unremarkable, and a set of blood cultures was sterile. A sputum culture yielded *Haemophilus influenzae* sensitive to ampicillin, and smears and cultures for mycobacteria in one stool and three sputum samples were negative.

The patient was treated with oral amoxicillin for 2 weeks without improvement. Empirical therapy against *M. avium* complex with clarithromycin, ciprofloxacin, and ethambutol was started; the patient's condition improved dramatically within the next few days, and the fever and diaphoresis disappeared, although cough and sputum production remained unchanged. Three weeks later, a slow-growing nonphotochromogenic mycobacterium, identified as *M. nonchromogenicum* in a reference laboratory (Centro Nacional de Microbiología, Majadahonda, Madrid, Spain) by biochemical tests and confirmed by polymerase chain reaction-restriction enzyme pattern analysis, was isolated from a blood sample obtained on admission. This microorganism was sensitive to the three drugs administered, and the treatment was continued. Two months later the patient had gained 10 kg, hemoglobin had increased to 13.8 g/dL, the erythrocyte sedimentation rate had decreased to 52 mm/h, and the differential leukocyte count had returned to normal. Antimycobacterial drugs were withheld after 1 year of treatment. Twenty-two months after the diagnosis, the patient is doing well. He is receiving combination antiretroviral therapy, and his CD4-cell count is 128/µL.

*M. nonchromogenicum*, a slow-growing non-pigmented (Runyon's group III) mycobacterium, belongs to the *M. terrae* complex, together with *M. triviale*; it is traditionally considered nonpathogenic. However, it has been involved in a few cases of pulmonary infection (12) and chronic tenosynovitis secondary to puncture wounds (13), like the related organism *M. terrae*. In fact, some authors think that *M. nonchromogenicum* is the true pathogen in the *M. terrae* complex (13), and it is possible that some reports have misidentified this organism. This complex was first isolated in soil washings from radishes, but it has been found to be ubiquitous in the aquatic environment, including a hospital potable water supply (14).

Unlike osteoarticular infections, which commonly occur in previously healthy people, the scanty reports on pulmonary and disseminated infection by *M. terrae* complex suggest that either immunosuppression or local predisposing conditions (e.g., tuberculous cavities) are necessary pathogenetic cofactors (15). To our knowledge, *M. nonchromogenicum* bacteremia has never been reported before.

No specific DNA probes exist for *M. terrae* complex, but false-positive reactions with *M. tuberculosis* complex DNA probes have been described (16). Isolates are usually resistant to most antituberculosis drugs, with the exception of ethambutol and streptomycin, and susceptible to erythromycin, ciprofloxacin, and sulfonamides.

Only one case of disseminated infection by *M. terrae* has been described in a patient with advanced HIV infection and positive cultures in blood and bronchoalveolar lavage fluid, but no additional data were provided (9). Although the isolate we recovered might represent a laboratory contaminant, several pieces of evidence make this possibility very unlikely: lack of alternative explanation for a persistent and progressive clinical picture of 4 months' duration, absence of response to standard antibiotic therapy, negative results in the search for other pathogens, rapid and sustained clinical and laboratory response to drugs active against this strain, clear improvement despite the lack of treatment for other conditions, and absence of other isolates of this pathogen in our hospital despite the large number of samples examined for mycobacteria.

### Acknowledgment

We are indebted to María Soledad Jiménez, Mycobacterial Laboratory of the Centro Nacional de Microbiología, Majadahonda, Madrid, for providing the microbiologic data.

**José Mayo, Julio Collazos, and Eduardo Martínez**

Hospital de Galdakao, Vizcaya, Spain

### References

1. Nightingale SD, Byrd LT, Southern PM, Jockusch JD, Cal SX, Wynne BA. Incidence of *Mycobacterium avium-intracellulare* complex bacteremia in human immunodeficiency virus-positive patients. J Infect Dis 1992;165:1082-5.
2. Raszka WV Jr, Skillman LP, McEvoy PL, Robb ML. Isolation of nontuberculous, non-avium mycobacteria from patients infected with human immunodeficiency virus. Clin Infect Dis 1995;20:73-6.
3. Bessesen MT, Shlay J, Stone-Venohr B, Cohn DL, Reves RR. Disseminated *Mycobacterium genavense* infection: clinical and microbiological features and response to therapy. AIDS 1993;7:1357-61.
4. Lessnau KD, Milanese S, Talavera W. *Mycobacterium gordonae*: a treatable disease in HIV-positive patients. Chest 1993;104:1779-85.
5. Straus WL, Ostroff SM, Jernigan DB, Kiehn TE, Sordillo EM, Armstrong D, et al. Clinical and epidemiologic characteristics of *Mycobacterium haemophilum*, an emerging pathogen in immunocompromised patients. Ann Intern Med 1994;120:118-25.
6. Parenti DM, Symington JS, Keiser J, Simon GL. *Mycobacterium kansasii* bacteremia in patients infected with human immunodeficiency virus. Clin Infect Dis 1995;21:1001-3.
7. Chocarro A, González-López A, Breznes MF, Canut A, Rodríguez J, Diego JM. Disseminated infection due to *Mycobacterium malmoense* in a patient infected with human immunodeficiency virus. Clin Infect Dis 1994;19:203-4.
8. Tchornobay AM, Claudy AL, Perrot JL, Levigne V, Denis M. Fatal disseminated *Mycobacterium marinum* infection. Int J Dermatol 1992;31:286-7.
9. Schafer RW, Sierra MF. *Mycobacterium xenopi*, *Mycobacterium fortuitum*, *Mycobacterium kansasii*, and other nontuberculous mycobacteria in an area of endemicity for AIDS. Clin Infect Dis 1992;15:161-2.
10. Valero G, Peters J, Jorgensen JH, Graybill JR. Clinical isolates of *Mycobacterium simiae* in San Antonio, Texas: an 11-yr review. Am J Respir Crit Care Med 1995;152:1555-7.
11. Roig P, Nieto A, Navarro V, Bernacer B, Borras R. Micobacteriosis por *Mycobacterium szulgai* en paciente con infección por el virus de la inmunodeficiencia humana. An Med Interna 1993;10:182-4.
12. Tsukamura M, Kita N, Otsuka W, Shimoide H. A study of the taxonomy of the *Mycobacterium nonchromogenicum* complex and report of six cases of lung infection due to *Mycobacterium nonchromogenicum*. Microbiol Immunol 1983;27:219-36.
13. Ridderhof JC, Wallace RJ Jr, Kilburn JO, Butler WR, Warren NG, Tsukamura M, et al. Chronic tenosynovitis of the hand due to *Mycobacterium nonchromogenicum*: use of high-performance liquid chromatography for identification of isolates. Rev Infect Dis 1991;13:857-64.
14. Lockwood WW, Friedman C, Bus N, Pierson C, Gaynes R. An outbreak of *Mycobacterium terrae* in clinical specimens associated with a hospital potable water supply. American Review of Respiratory Diseases 1989;140:1614-7.
15. Peters EJ, Morice R. Miliary pulmonary infection caused by *Mycobacterium terrae* in an autologous bone marrow transplant patient. Chest 1991;100:1449-50.
16. Ford EG, Snead SJ, Todd J, Warren NG. Strains of *Mycobacterium terrae* complex which react with DNA probes for *M. tuberculosis* complex. J Clin Microbiol 1993;31:2805-6.

